# Excess risk of hospitalisation for heart failure among people with type 2 diabetes

**DOI:** 10.1007/s00125-018-4700-5

**Published:** 2018-08-09

**Authors:** Annika Rosengren, Jon Edqvist, Araz Rawshani, Naveed Sattar, Stefan Franzén, Martin Adiels, Ann-Marie Svensson, Marcus Lind, Soffia Gudbjörnsdottir

**Affiliations:** 1 Department of Molecular and Clinical Medicine, Institute of Medicine, University of Gothenburg, Sahlgrenska University Hospital, SE 413 45 Gothenburg, Sweden; 2 Sahlgrenska University Hospital, Östra Hospital, Gothenburg, Sweden; 3 Institute of Cardiovascular and Medical Sciences, BHF Glasgow Cardiovascular Research Centre, University of Glasgow, Glasgow, UK; 4 Swedish National Diabetes Register, Centre of Registers, Gothenburg, Sweden; 5 Health Metrics Unit, Sahlgrenska Academy, University of Gothenburg, Gothenburg, Sweden; 6 Department of Medicine, NU Hospital Group, Uddevalla, Sweden

**Keywords:** Albuminuria, HbA_1c_, Heart failure, Registries, Type 2 diabetes

## Abstract

**Aims/hypothesis:**

Type 2 diabetes is an established risk factor for heart failure, but age-specific data are sparse. We aimed to determine excess risk of heart failure, based on age, glycaemic control and kidney function in comparison with age- and sex-matched control individuals from the general population.

**Methods:**

Individuals with type 2 diabetes registered in the Swedish National Diabetes Registry 1998–2012 (*n* = 266,305) were compared with age-, sex- and county-matched control individuals without diabetes (*n* = 1,323,504), and followed over a median of 5.6 years until 31 December 2013.

**Results:**

We identified 266,305 individuals with type 2 diabetes (mean age 62.0 years, 45.3% women) and 1,323,504 control individuals. Of the individuals with type 2 diabetes and control individuals, 18,715 (7.0%) and 50,157 (3.8%) were hospitalised with a diagnosis of heart failure, respectively. Comparing individuals with diabetes with those in the control group, men and women with type 2 diabetes who were younger than 55 years of age had HRs for hospitalisation for heart failure of 2.07 (95% CI 1.73, 2.48) and 4.59 (95% CI 3.50, 6.02), respectively, using analyses adjusted for socioeconomic variables and associated conditions. Younger age, poorer glycaemic control and deteriorating renal function were all associated with increased excess risk of heart failure in those with type 2 diabetes compared with the control group. However, people with diabetes who were ≥75 years and without albuminuria or with good glycaemic control (HbA_1c_ ≤52 mmol/mol [≤6.9%]) had a similar risk of hospitalisation for heart failure as control individuals in the same age group.

**Conclusions/interpretation:**

Men and women aged <55 years with type 2 diabetes are at markedly elevated excess risk of heart failure. The excess risk declined with age, but persisted even with good glycaemic control. However, among those who were 75 years and older, diabetic individuals with well controlled glucose levels or without albuminuria had a risk of heart failure that was on a par with individuals without diabetes.

**Electronic supplementary material:**

The online version of this article (10.1007/s00125-018-4700-5) contains peer-reviewed but unedited supplementary material, which is available to authorised users.

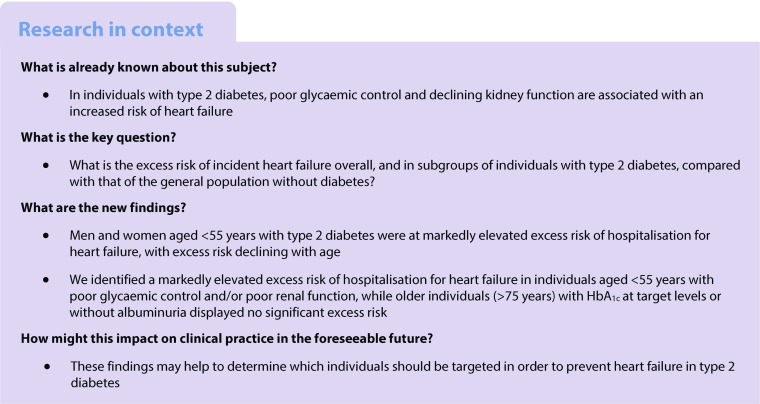



## Introduction

The study of the role of type 2 diabetes in the development of heart failure is somewhat neglected [1] but it is increasingly being recognised [1, 2]. Heart failure has been shown to be among the most common initial manifestations of cardiovascular disease in type 2 diabetes [3, 4]; there is a strong correlation between poor glycaemic control among individuals with type 2 diabetes and risk of heart failure [4, 5]. This is clinically important because, if shown to be causal, improved glycaemic control could decrease the risk of developing heart failure among people with diabetes, although it is becoming increasingly clear that the mode of glucose reduction is also critical to heart-failure risk [6].

Incidence and prevalence of heart failure and type 2 diabetes both increase with age. Accordingly, most of what we know about type 2 diabetes as a risk factor for heart failure, and about predictors of heart failure in the population with diabetes, is based on studies in people of middle age and older. Prior studies, using data collected until 1996, have identified diabetes as a strong predictor of heart failure, particularly in younger individuals [7, 8]. However, these studies have been limited in size and, furthermore, prognosis with respect to cardiovascular outcomes in type 2 diabetes has considerably improved in recent years [9]. Hence, there is a need for updated information with respect to age-specific risk of heart failure in type 2 diabetes and predictors of heart failure in this population. To this end, we created a large dataset by combining detailed clinical information on adults (men and women) with type 2 diabetes from the Swedish National Diabetes Register (NDR), with data from age-, sex- and county-matched individuals without diabetes (control group) from the population registry. Outcome data on hospitalisation for heart failure was available from the hospital registry. The aims of the study were to estimate age-specific excess risk in people with and without type 2 diabetes and to identify predictors of excess risk of hospitalisation for heart failure within the diabetic population.

## Methods

### Study population

Individuals with at least one registration in the NDR from 1 January 1998 until 31 December 2012 were eligible for inclusion in the study. For each individual, five control individuals, matched for age (by birth year), sex and county, were randomly selected from the general population in Sweden [10]. The cohort consisted of 457,473 individuals with type 2 diabetes and 2,287,365 matched control individuals. The study was approved by the ethics review board at the University of Gothenburg and informed consent was obtained from each individual registered in the NDR. All personal identifiers were removed from the combined dataset before analysis.

**Fig. 1:**
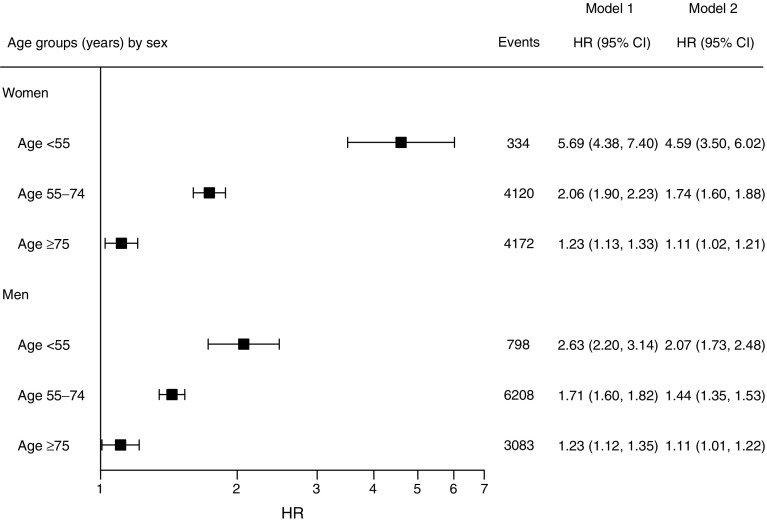
HR (95% CI) for the risk of hospitalisation for heart failure among individuals with type 2 diabetes by sex and age group, compared with age- and sex-matched control individuals from the general population. Model 1 shows HRs adjusted for age and duration of diabetes. Model 2 shows HRs adjusted for age, duration of diabetes, income, education, marital status, immigration status, stroke, acute myocardial infarction, coronary heart disease, atrial fibrillation and renal dialysis or transplantation. The *x*-axis is plotted on a log_*e*_ scale. Events are presented as numbers (*n*). Black boxes indicate HRs using Model 2, while error bars indicate 95% CIs. Plots for Model 1 are not shown

**Fig. 2:**
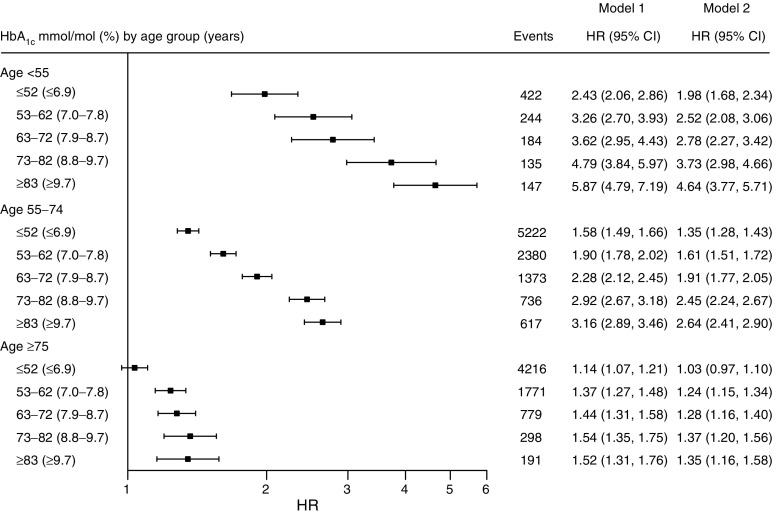
HR (95% CI) for the risk of hospitalisation for heart failure among individuals with type 2 diabetes by HbA_1c_ in mmol/mol (%) by age group, compared with age- and sex-matched control individuals from the general population. Model 1 shows HRs adjusted for age, sex and duration of diabetes. Model 2 shows HRs adjusted for age, sex, duration of diabetes, income, education, marital status, immigration status, stroke, acute myocardial infarction, coronary heart disease, atrial fibrillation and renal dialysis or transplantation. The *x*-axis is plotted on a log_*e*_ scale. Events are presented as numbers (*n*). Black boxes indicate HRs using Model 2, while error bars indicate 95% CIs. Plots for Model 1 are not shown

**Fig. 3:**
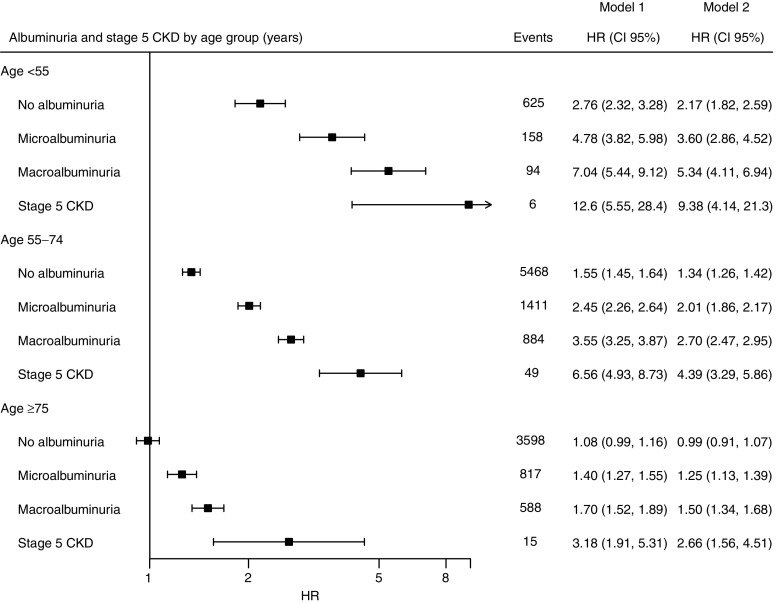
HR (95% CI) for the risk of hospitalisation for heart failure among individuals with type 2 diabetes by albuminuria and stage 5 CKD by age group, compared with age- and sex-matched control individuals from the general population. Model 1 shows HRs adjusted for age, sex and duration of diabetes. Model 2 shows HRs adjusted for age, sex, duration of diabetes, income, education, marital status, immigration status, stroke, acute myocardial infarction, coronary heart disease and atrial fibrillation. The *x*-axis is plotted on a log_*e*_ scale. Events are presented as numbers (*n*). Black boxes indicate HRs using Model 2, while error bars indicate 95% CIs. Plots for Model 1 are not shown. 5002 events are missing because of missing data on albuminuria and stage 5 CKD

A flow chart of the selection procedure is presented in electronic supplementary material [ESM] Fig. 1 (this selection procedure was used to generate data in Tables 1–2, Figs 1–3, ESM Tables 2–7 and ESM Figs 2–6). We excluded control individuals with inconsistent vital data, individuals (both with and without diabetes) with previous heart failure (either as a primary [principal] diagnosis or a contributory diagnosis; see ESM Table 1 for ICD-9 [www.icd9data.com/2007/Volume1] and ICD-10 [http://apps.who.int/classifications/icd10/browse/2016/en] codes; 428 [ICD-9] and I50 [ICD-10] were used to determine hospitalisation for heart failure for the purposes of exclusion) and individuals with diabetes where information on diabetes duration was missing. In the NDR, diabetes duration was recorded as the time from being diagnosed until first registration in the NDR and, accordingly, some individuals may have had type 2 diabetes for several years before registration, without treatment. In order to obtain a cohort representative of current management of type 2 diabetes, we therefore excluded 55,842 individuals with diabetes with a duration of diabetes >10 years, and their matched controls. We imputed missing data for HbA_1c_, albuminuria and eGFR. Imputation was done using first value carried backward because of the high within-patient correlation for these variables. We restricted the imputation to values occurring within 365 days of the index date, provided that the individual had not experienced heart failure or any intervening event (coronary heart disease, stroke, myocardial infarction, atrial fibrillation, renal dialysis or transplantation) during that period. HbA_1c_ was missing for 49,103 individuals with diabetes before imputation; after imputation and applied exclusion criteria, 9963 had missing HbA_1c_ and were excluded along with their matched controls. Finally, 266,305 individuals with type 2 diabetes and 1,323,504 matched control individuals were included in the analysis. From Statistics Sweden, we retrieved information on income, education (categorised as ≤ years, 10 to 12 years, or college/university degree) and country of birth (categorised as Sweden or other) for individuals with diabetes and controls.

**Table 1:** Baseline characteristics of individuals with type 2 diabetes by categories of HbA_1c_, and of control individuals matched for age, sex and county

Variable^a^	Control individuals	Individuals with T2D	HbA_1c_ ≤52 mmol/mol (≤6.9%)	HbA_1c_ 53–62 mmol/mol (7.0–7.8%)	HbA_1c_ 63–72 mmol/mol (7.9–8.7%)	HbA_1c_ 73–82 mmol/mol (8.8–9.7%)	HbA_1c_ ≥83 mmol/mol (≥9.7%)
Individuals^b^, *n*	1,323,504	266,305	158,091	53,731	26,558	13,077	14,848
Age and sex							
Women, *n* (%)	599,742 (45.3)	120,641 (45.3)	74,560 (47.2)	24,095 (44.8)	11,162 (42.0)	5252 (40.2)	5572 (37.5)
Age years	61.9 (11.6)	62.0 (11.6)	62.7 (11.5)	62.4 (11.4)	60.7 (11.7)	58.9 (11.7)	57.2 (11.5)
Socioeconomic status							
Swedish born, *n* (%)	1,156,415 (87.4)	217,396 (81.6)	131,296 (83.1)	43,488 (80.9)	21,089 (79.4)	10,263 (78.5)	11,260 (75.8)
Marital status, *n* (%)							
Divorced	219,483 (16.6)	45,970 (17.3)	26,198 (16.6)	9378 (17.5)	4900 (18.5)	2477 (18.9)	3017 (20.3)
Married	757,676 (57.2)	145,885 (54.8)	88,961 (56.3)	29,350 (54.6)	13,918 (52.4)	6627 (50.7)	7029 (47.3)
Single	219,019 (16.5)	46,110 (17.3)	25,541 (16.2)	8909 (16.6)	5046 (19.0)	2830 (21.6)	3784 (25.5)
Widowed	127,262 (9.6)	28,340 (10.6)	17,391 (11.0)	6094 (11.3)	2694 (10.1)	1143 (8.7)	1018 (6.9)
Education, *n* (%)							
≤9 years	424,310 (32.5)	104,051 (39.8)	60,943 (39.1)	21,955 (41.7)	10,723 (41.3)	5133 (40.3)	5297 (36.5)
10–12 years	542,668 (41.5)	112,736 (43.1)	66,869 (42.9)	22,407 (42.5)	11,160 (43.0)	5597 (43.9)	6703 (46.2)
College/university	339,790 (26.0)	44,903 (17.2)	27,985 (18.0)	8310 (15.8)	4090 (15.7)	2013 (15.8)	2505 (17.3)
Disposable income (SEK^c^), median (interquartile range)	1652 (1163–2425)	1453 (1091–2114)	1470 (1109–2134)	1416 (1067–2071)	1408 (1054–2030)	1425 (1056–2081)	1506 (1088–2231)
Coexisting conditions at baseline, *n* (%)							
Atrial fibrillation	35,645 (2.7)	11,078 (4.2)	6932 (4.4)	2234 (4.2)	1025 (3.9)	497 (3.8)	390 (2.6)
Acute myocardial infarction	36,703 (2.8)	16,765 (6.3)	10,218 (6.5)	3548 (6.6)	1656 (6.2)	720 (5.5)	623 (4.2)
Coronary heart disease	77,025 (5.8)	32,637 (12.3)	19,896 (12.6)	6973 (13.0)	3213 (12.1)	1373 (10.5)	1182 (8.0)
Stroke	36,059 (2.7)	12,262 (4.6)	7661 (4.8)	2499 (4.7)	1172 (4.4)	498 (3.8)	432 (2.9)
Renal dialysis or transplantation	1334 (0.1)	394 (0.1)	238 (0.2)	83 (0.2)	44 (0.2)	12 (0.1)	17 (0.1)
Cancer	74,773 (5.6)	16,814 (6.3)	10,386 (6.6)	3453 (6.4)	1613 (6.1)	665 (5.1)	697 (4.7)
Variables from the NDR							
Diabetes duration (years)	NA	2.8 (3.0)	2.4 (2.7)	3.4 (3.2)	3.7 (3.3)	3.6 (3.4)	2.4 (3.2)
Debut age of diabetes (years)	NA	59.2 (11.5)	60.3 (11.4)	59.0 (11.3)	57.0 (11.5)	55.4 (11.5)	54.8 (11.6)
HbA_1c_ (mmol/mol)	NA	53.8 (15.2)	44.6 (5.0)	56.5 (2.6)	66.8 (2.8)	76.9 (2.8)	98.1 (13.0)
HbA_1c_ (%)	NA	7.1 (1.39)	6.2 (0.46)	7.3 (0.24)	8.3 (0.26)	9.2 (0.26)	11.1 (1.19)
LDL-cholesterol (mmol/l)	NA	3.0 (1.0)	3.0 (0.9)	3.0 (1.0)	3.0 (1.0)	3.1 (1.0)	3.3 (1.1)
Total cholesterol (mmol/l)	NA	5.1 (1.1)	5.1 (1.1)	5.1 (1.1)	5.2 (1.1)	5.3 (1.2)	5.6 (1.4)
Smokers, *n* (%)	NA	41,537 (17.0)	22,818 (15.7)	8447 (17.1)	4669 (19.2)	2571 (21.5)	3032 (22.7)
BMI (kg/m^2^)	NA	30.2 (5.5)	29.9 (5.3)	30.5 (5.5)	30.6 (5.6)	31.0 (5.9)	30.6 (6.0)
Systolic BP (mmHg)	NA	138.9 (17.6)	138.2 (17.3)	139.9 (17.8)	140.2 (18.1)	140.3 (18.6)	139.0 (18.7)
Diastolic BP (mmHg)	NA	79.6 (9.7)	79.0 (9.5)	79.9 (9.7)	80.6 (9.7)	81.4 (10.0)	82.3 (10.3)
Albuminuria, *n* (%)	NA						
No albuminuria	NA	159,089 (81.8)	97,256 (84.2)	31,816 (80.4)	15,170 (77.6)	7172 (75.5)	7675 (74.8)
Microalbuminuria	NA	24,436 (12.6)	12,839 (11.1)	5299 (13.4)	2983 (15.3)	1580 (16.6)	1735 (16.9)
Macroalbuminuria	NA	10,929 (5.6)	5472 (4.7)	2460 (6.2)	1404 (7.2)	742 (7.8)	851 (8.3)
eGFR (ml min^−1^ [1.73 m] ^−2^)	NA	84.3 (23.0)	82.2 (21.5)	84.3 (23.1)	87.4 (24.6)	91.0 (26.0)	97.1 (27.5)
Antihypertensive therapy, *n* (%)	NA	153,251 (61.5)	94,757 (63.9)	31,267 (62.1)	14,390 (57.9)	6507 (53.5)	6330 (47.1)
Statin therapy, *n* (%)	NA	99,690 (40.0)	61,013 (41.1)	20,876 (41.4)	9476 (38.3)	4276 (35.3)	4049 (30.2)
Glucose-lowering therapy, *n* (%)	NA						
No pharmacological treatment	NA	104,635 (39.3)	82,506 (52.2)	13,920 (25.9)	4234 (15.9)	1726 (13.2)	2249 (15.1)
Oral agents	NA	120,556 (45.3)	60,650 (38.4)	29,949 (55.7)	14,884 (56.0)	7110 (54.4)	7963 (53.6)
Insulin	NA	21,252 (8.0)	8529 (5.4)	4845 (9.0)	3598 (13.5)	2072 (15.8)	2208 (14.9)
Insulin + oral agents	NA	19,862 (7.5)	6406 (4.1)	5017 (9.3)	3842 (14.5)	2169 (16.6)	2428 (16.4)

Data are presented as mean (SD), unless otherwise stated

^a^Categories of HbA_1c_ were defined based on mmol/mol (IFCC) data. For exact category levels in % (DCCT) use the conversion formula, DCCT = 0.09148 × IFCC + 2.152

^b^Variables that do not sum up to the overall number of individuals contain missing data and are not included

^c^Annual disposable income is given in hundred Swedish kronor (SEK)

NA, not available; T2D, type 2 diabetes

**Table 2:** Hospitalisation for heart failure

Group	Heart failure events (any diagnosis), *n*	Event rate per 1000 person-years (95% CI)	Heart failure events (primary or secondary diagnosis^a^), *n*	Event rate per 1000 person-years (95% CI)
Overall				
Controls	50,157	6.2 (6.2, 6.3)	22,259	2.7 (2.7, 2.8)
T2D	18,715	11.9 (11.8, 12.1)	9326	5.9 (5.7, 6.0)
Individuals with T2D by HbA_1c_ [mmol/mol (%)]				
≤52 (≤6.9)	9860	10.8 (10.6, 11.0)	4784	5.2 (5.0, 5.3)
53–62 (7.0–7.8)	4395	13.4 (13.0, 13.8)	2204	6.6 (6.3, 6.9)
63–72 (7.9–8.7)	2336	13.8 (13.2, 14.3)	1208	7.0 (6.6, 7.4)
73–82 (8.8–9.7)	1169	14.5 (13.7, 15.4)	599	7.3 (6.7, 7.9)
≥83 (≥9.7)	955	12.5 (11.8, 13.4)	531	6.9 (6.3, 7.5)
Individuals with T2D by albuminuria and stage 5 CKD^b^				
No albuminuria	9691	9.9 (9.7, 10.1)	4769	4.8 (4.7, 5.0)
Microalbuminuria	2386	16.4 (15.7, 17.0)	1269	8.5 (8.1, 9.0)
Macroalbuminuria	1566	25.4 (24.1, 26.7)	821	12.9 (12.1, 13.9)
Stage 5 CKD	70	31.9 (24.9, 40.3)	28	12.3 (8.2, 17.8)
Individuals with T2D by eGFR (ml min^−1^ [1.73 m] ^−2^) and stage 5 CKD^b^				
>90	2511	6.3 (6.1, 6.6)	1173	2.9 (2.8, 3.1)
60–90	6428	10.1 (9.9, 10.3)	3169	4.9 (4.7, 5.1)
45–60	2604	21.1 (20.3, 21.9)	1294	10.2 (9.7, 10.8)
30–45	989	41.4 (38.9, 44.1)	513	20.6 (18.8, 22.4)
15–30	219	65.9 (57.5, 75.2)	123	35.2 (29.2, 42.0)
Stage 5 CKD	70	31.9 (24.9, 40.3)	28	12.3 (8.2, 17.8)

95% CI were estimated by exact Poisson confidence limits

^a^Primary or first contributory (secondary) diagnosis

^b^Individuals with diabetes who had missing data on albuminuria or eGFR were excluded in the respective analyses. Numbers missing; *n* = 5002 for albuminuria and Stage 5 CKD, *n* = 5894 for eGFR and Stage 5 CKD

T2D, type 2 diabetes

HbA_1c_ is given as mmol/mol according to the International Federation of Clinical Chemistry and converted into per cent according to the Diabetes Control and Complications Trial. Microalbuminuria was defined as two positive results for three urine samples obtained within 1 year of the first registration, with positivity defined as an albumin/creatinine ratio of 3–30 mg/mmol (approximately 30–300 mg/g) or a urinary albumin clearance of 20–200 μg/min (20–300 mg/l). Macroalbuminuria was defined as an albumin/creatinine ratio of more than 30 mg/mmol (approximately ≥300 mg/g) or a urinary albumin clearance of >200 μg/min (>300 mg/l). The eGFR was calculated by means of the Modification of Diet in Renal Disease equation [11]. Stage 5 chronic kidney disease (CKD) was defined as the need for renal dialysis or renal transplantation or as an eGFR of less than 15 ml min^−1^ [1.73 m] ^−2^.

### Coexisting conditions and outcomes

Information on coexisting conditions and outcomes was obtained by linkage to the nationwide Swedish Inpatient and Cause-Specific Mortality registries for individuals with diabetes and controls. ICD-9 and -10 codes have been used to define acute myocardial infarction, coronary heart disease, hospitalisation for heart failure, atrial fibrillation, stroke, renal dialysis or transplantation (ESM Table 1). Major cardiovascular diagnoses in the Swedish Hospital Register, including heart failure, have been validated and found to have high positive predictive values for these diagnoses [12, 13].

Individuals with type 2 diabetes and control individuals were followed until first hospitalisation with heart failure as a primary or contributory diagnosis, until death, or until 31 December 2013. We compared the age- and sex-specific risk of hospitalisation for heart failure among individuals with type 2 diabetes with the risk in matched control individuals, according to HbA_1c_ levels, albuminuria (categorised as normoalbuminuria, microalbuminuria, macroalbuminuria or stage 5 CKD) and eGFR levels (categorised as >90 ml min^−1^ [1.73 m] ^−2^, >60–90 ml min^−1^ [1.73 m] ^−2^, >45–60 ml min^−1^ [1.73 m] ^−2^, >30–45 ml min^−1^ [1.73 m] ^−2^, 15–30 ml min^−1^ [1.73 m] ^−2^, or stage 5 CKD [includes renal dialysis or transplantation or eGFR <15 ml min^−1^ [1.73 m] ^−2^]).

### Statistical methods

Crude mortality rates are described as events per 1000 person-years, with 95% exact Poisson CIs. Survival analyses were performed using Cox regression. To study the association between HbA_1c_ and risk of heart failure, participants were analysed in three separate age categories (<55 years, 55–74 years and ≥75 years; baseline data for the respective age group are presented in ESM Table 2). We also analysed the association between albuminuria, eGFR and heart failure by age group. Model 1 was adjusted for age, sex and duration of diabetes. Model 2 was additionally adjusted for income, education, immigration status and status at baseline with regard to a history of coexisting conditions (acute myocardial infarction, coronary heart disease, atrial fibrillation, stroke, and renal dialysis or transplantation). We performed a subgroup analysis restricted to individuals with diabetes (and matched control individuals) with normoalbuminuria. Since we excluded 96,127 individuals with diabetes because of missing information on diabetes duration or because duration of diabetes was >10 years, we performed sensitivity analyses, where we examined participants without excluding diabetic individuals (and control individuals) because of diabetes duration (see ESM Fig. 7 for a flow chart of the selection procedure used to generate data for ESM Figs 8–11).

In the analyses examining the relationship between renal variables and heart failure, we did not adjust for presence of renal dialysis or transplantation at baseline. Individuals in the control group were assigned to a diabetes duration of 0 years to omit the effect of duration on the hazard estimates for control individuals, while still allowing for correct modelling of the association between duration and outcomes among individuals with diabetes. For individuals with type 2 diabetes, main Cox models were stratified on diabetes duration, which ranged from 0 to 10 years. In models including individuals with >10 years’ duration of diabetes (and controls), duration was centred around the population grand mean instead; thus, the HRs represent the adjusted risks at the mean duration of diabetes (~5.1 years).

Throughout, only 1–5% of observations were omitted from the models because of missing data on covariates. All the analyses were performed with R (R Foundation for Statistical Programming, www.r-project.org) version 3.2.1.

## Results

### Participant characteristics

By design, age and sex were equal among the diabetic and control groups (mean 62 years, 45% women; see Table 1 and the flow chart for the final cohort in ESM Fig. 1). A lower proportion of individuals with diabetes had a college/university degree (17.2% vs 26.0%) and fewer were born in Sweden (81.6% vs 87.4%). Among individuals with diabetes, mean duration of diabetes was 2.8 years (SD 3.0 years). Those with high HbA_1c_ values were younger, more frequently smokers, had a greater degree of albuminuria, but higher eGFR, and lower rates of statin and antihypertensive treatment.

#### Incidence rates and HRs by sex, age, glycaemic control and renal function

Over a median follow-up of 5.6 years, 18,715/266,305 (7.0%) of individuals with diabetes and 50,157/1,323,504 (3.8%) of control individuals were hospitalised with any diagnosis of heart failure, either as a primary/principal or contributory diagnosis. Incidence rates of heart failure hospitalisations were 11.9 and 6.2 per 1000 person-years for diabetic individuals and control individuals, respectively (Table 2). If only heart failure as a main, or as the first contributory (secondary) diagnosis was considered, the corresponding rates were 5.9 and 2.7 per 1000 person-years, respectively. Baseline characteristics for risk factors by age group are presented in ESM Table 2.

Absolute age-specific risks for heart failure (as a primary or any contributory diagnosis) were higher in men with diabetes than in women with diabetes, increasing from 3.2 per 1000 person-years in men <55 years to 43.7 per 1000 person-years in men ≥75 years, and 2.1 per 1000 person-years in women <55 years to 34.3 per 1000 person-years in women ≥75 years (ESM Table 3).

As compared with control individuals, HRs for the risk of hospitalisation for heart failure after adjustment for age, sex, duration of diabetes, income, education, marital status, immigration status, stroke, acute myocardial infarction, coronary heart disease, atrial fibrillation and renal dialysis or transplantation for women and men aged less than 55 years were 4.59 (95% CI 3.50, 6.02) and 2.07 (95% CI 1.73, 2.48), respectively (Fig. 1). The excess risk was attenuated with increasing age.

Hospitalisations for heart failure rose with increasing HbA_1c_, irrespective of whether the heart failure diagnosis was as a primary or contributory condition (ESM Table 4, ESM Fig. 2). The excess risk for heart failure was considerably higher among younger individuals with diabetes with a monotonic risk increase with higher HbA_1c_ in all age groups. After adjustment for age, sex, duration of diabetes, income, education, marital status, immigration status, stroke, acute myocardial infarction, coronary heart disease, atrial fibrillation and renal dialysis or transplantation, the youngest individuals with diabetes had a 2.0–4.6-fold higher risk, whereas the oldest diabetic individuals with HbA_1c_ below the target level of ≤52 mmol/mol (6.9%) displayed no significant excess risk of hospitalisation for heart failure (Fig. 2). Overall HRs are presented in ESM Fig. 2.

Deteriorating renal function, whether defined as micro- or macroalbuminuria (ESM Fig. 3) or as decreasing eGFR (ESM Fig. 4), was associated with excess risk of hospitalisation for heart failure; this association was less pronounced with increasing age (Fig. 3 and ESM Fig. 5). The relationship between eGFR and hospitalisation for heart failure was, however, slightly J-shaped with eGFR >60–90 ml min^−1^ [1.73 m] ^−2^ displaying the lowest excess risk (ESM Fig. 4). Incidence rates by age and indicators of renal function are shown in ESM Tables 5 and 6.

In a further step, we restricted our analysis of the association between glycaemic control and heart failure to diabetic individuals without albuminuria (*n* = 159,089). In the fully adjusted model, individuals with diabetes who were younger than 75 years with HbA_1c_ within target still had a higher excess risk compared with control individuals (ESM Fig. 6). Among participants aged <55 years, adjusted HRs increased from 1.70 (95% CI 1.36, 2.12) to 4.05 (95% CI 3.03, 5.40), from those with the best to the poorest glycaemic control.

In ESM Table 7, the characteristics for the individuals with diabetes with missing HbA_1c_ data, presented in the flow chart (ESM Fig. 1), are displayed alongside the characteristics of the overall cohort included in the study.

A sensitivity analysis (second flow-chart for sensitivity analyses in ESM Fig. 7), which included individuals with missing information on diabetes duration at baseline and those with diabetes duration >10 years (ESM Figs 8–11), displayed largely the same associations as with our original cohort, but with slightly higher relative risk estimates.

## Discussion

In this nationwide study of 266,305 individuals with type 2 diabetes and population-based age- and sex-matched control individuals, excess risk of hospitalisation for heart failure among people with diabetes varied from mildly elevated to markedly high excess risk, particularly in younger individuals, especially younger women, and in those with poor glycaemic control, and/or impaired renal function.

Young people (<55 years) with good glycaemic control experienced a twofold excess risk, whereas those with poor glycaemic control experienced a 4.6-fold excess risk of hospitalisation for heart failure compared with control individuals. There was a successive attenuation of risk with increasing age, such that older diabetic individuals (≥75 years) with good glycaemic control experienced no (or only a slight) excess risk, and no excess risk at all in the absence of albuminuria. Similarly, there was a steady increase in excess risk with declining kidney function, whether measured as decreasing eGFR or micro- or macroalbuminuria. Here, too, the excess risk was attenuated with increasing age.

Young women with diabetes experienced an almost fivefold increased risk of hospitalisation for heart failure, while there was a twofold excess risk among young men: a greater excess risk than that seen for cardiovascular disease mortality [7]. This was due to the overall low absolute risk in women below 55 years in the general population, but the absolute rates in women with diabetes of this age group were also lower than in men with diabetes. Absolute risk was higher in men at any age, in diabetic individuals as well as control individuals. Sex differences in both absolute and relative risks decreased with age. Both diabetes and male sex are known to be associated with increased risk of heart failure [2, 14, 15]. Our findings are in accordance with cohort studies that have demonstrated that diabetes increases the risk of heart failure more in women than in men [16]. We are not aware of any study, however, that has quantified age-specific relative risks for heart failure associated with diabetes in men and women. Further work is needed to explain the higher relative risk for heart failure in women compared with men with diabetes. Of interest, in order to develop diabetes, women have to put on more weight than do men, and obesity is also a risk factor for heart failure [17].

In a previous study, we found that the excess risk of death and cardiovascular death did not persist among older individuals with type 2 diabetes after adjusting for confounders, which in part implies that preventive strategies may have succeeded to halt premature death in a large proportion of the diabetic population [10]; but, even so, diabetes appears to be a much more toxic condition in the young. However, when considering older individuals with diabetes, survival bias must be kept in mind, where people with a very long duration of diabetes might not survive to advanced age. Still, because we restricted our sample to individuals with a diabetes duration of <10 years, this effect would have been lessened. Our current study, however, shows that diabetes confers a near-ubiquitous but varying excess risk of heart failure in all age groups and at all levels of glycaemic control; however, not in older people in the absence of albuminuria, or whose blood glucose is well controlled. This adds important knowledge to the increasing body of evidence suggesting that heart failure is a diabetes-related complication, and adds information to prior findings of high rates of heart failure in cohorts with type 2 diabetes in the UK and the USA [3, 18].

In the group with diabetes, those with good glycaemic control were less often smokers and more frequently treated with antihypertensives, but had lower eGFR. Judging from the characteristics of the groups, we did not find any obvious confounders that might invalidate the excess risk of hospitalisation for heart failure associated with poor glycaemic control. As a group, control individuals should have had lower levels of risk factors, meaning that the independent effect of diabetes might have been overestimated. In a previous study including only people with type 2 diabetes, we showed that glycaemic control was an independent predictor of developing heart failure, even after adjusting for an array of covariates [4]. We have also demonstrated the same association between blood glucose and risk of hospitalisation for heart failure among people with type 1 diabetes [19], who, as a group, do not display the same obesity-related disorders as people with type 2 diabetes.

Heart failure is a common consequence of type 2 diabetes [2]. The association is caused by the role of diabetes as a risk factor for coronary heart disease, as well as by the often-coexisting hypertension and obesity that might accelerate heart failure, and the potential direct effects of diabetes on the myocardium. This apparent ‘diabetic cardiomyopathy’ may arise from multiple causes, including disease of the interstitium (e.g. fibrosis), as well as direct myocardial effects, including disturbances of glucose and fatty acid metabolism [1].

Given that heart failure is common and is associated with poor prognosis among individuals with diabetes [20], effective therapies are much needed. However, in a meta-analysis, no benefit on heart failure was observed with intensive vs relaxed glucose control (although these studies used older glucose-lowering drugs [21]), and neither has any glucose-lowering medication proven effective in preventing development of heart failure. However, the Empagliflozin, Cardiovascular Outcome Event Trial in Type 2 Diabetes Mellitus Patients (EMPA-REG OUTCOME) study reported that empagliflozin, which promotes excretion of glucose (and sodium) by inhibiting sodium–glucose cotransporter 2 (SGLT-2) in the kidneys, brought about a 34% reduction in the risk of hospitalisation for heart-failure [6].

The strength of the present study, in addition to having age- and sex-matched control individuals from the general population, lies in the longitudinal design, large size and national coverage of the study population, near-complete ascertainment for heart-failure hospitalisations and the presence of important covariates. To avoid possible bias in the estimation of excess risks from older management strategies and to provide data applicable to the current treatment of diabetes, we excluded individuals with diabetes duration >10 years before registration in the NDR, still generating a uniquely large sample of individuals with type 2 diabetes. However, there are also a few limitations to this study; we only assessed hospital admissions for heart failure, which means that milder cases managed in an outpatient setting were not included. However, heart-failure hospitalisation represents a well-validated and specific outcome [12, 13], and potential misclassification of heart-failure diagnoses would likely not vary by diabetes status or glycaemic control. Moreover, we did not have data on several important risk factors for the control individuals, such as blood pressure and body weight; these would have been desirable since both high BMI and hypertension lie on the causal pathway to heart failure.

In conclusion, we found that younger men and women with type 2 diabetes are at markedly high excess risk of hospitalisation for heart failure compared with age- and sex-matched control individuals. The excess risk declines with age, but persists in all age groups; however, it does not apply to the fairly large subgroup of individuals aged 75 and older with HbA_1c_ at target level or without albuminuria. In all other individuals, the risk increases markedly with poor glycaemic control and impaired renal function. In Sweden, the prevalence of diabetes is still low at about 6% in adults [22], but given the steep rise in type 2 diabetes worldwide, diabetes could contribute increasingly to the global burden of early-onset heart failure.

## Electronic supplementary material

ESM (PDF 1706 kb)

## Data Availability

The data that we have acquired from a combination of registers cannot be shared, but they are accessible, after relevant permissions from an ethics board and application to the registers in question.

## Abbreviations

CKD: Chronic kidney disease
NDR: Swedish National Diabetes Registry
