# Supplementary material for: Excess risk of hospitalisation for heart failure among people with type 2 diabetes
Source: Diabetologia. 2018 Aug 9;61(11):2300–9. doi: 10.1007/s00125-018-4700-5 (PMC6182656; doi:10.1007/s00125-018-4700-5)
Supplement: Supplementary file 1 — (PDF 1706 kb) [file 125_2018_4700_MOESM1_ESM.pdf]

Electronic supplementary material

Article: Age-specific excess risk of hospitalization for heart failure among persons with type 2 diabetes

***ESM Table 1*: Codes from the International Classification of Diseases 9th and 10th Revision. Used to Collect Coexisting Conditions and Outcomes**

- Coronary heart disease: 410-414 (ICD-9), I20-I25 (ICD-10)
- Acute myocardial infarction: 410 (ICD-9), I21 (ICD-10)
- Stroke: 431-434, 436 (ICD-9), I61-I64 (ICD-10)
- Hospitalization for heart failure: 428 (ICD-9), I50 (ICD-10)
- Atrial fibrillation: 427D (ICD-9), I48 (ICD-10).
- Renal dialysis and transplantation: V42A, V45B, V56A, V56W (ICD-9) and Z94.0, Z49, Z99.2, (ICD-10)

Note that in the analysis examining the excess risk of heart failure in relation to kidney function and kidney complications, we also assessed the patient's estimated glomerular filtration rate, which is available in the National Diabetes Register.

ESM Table 2: Baseline characteristics of patients with type 2 diabetes by age group and Hba$_{1c}$

Patients <55 years of age

	**Patients overall**	**≤52 mmol/mol (≤6.9%)**	**53-62 mmol/mol (7.0-7.8%)**	**63-72 mmol/mol (7.9-8.7%)**	**73-82 mmol/mol (8.8-9.7%)**	**≥83 mmol/mol (≥9.7%)**
Age (years) - mean (SD)	46.7 (6.6)	46.6 (6.6)	47.1 (6.3)	46.9 (6.5)	46.7 (6.7)	46.1 (6.9)
Diabetes duration (years) - mean (SD)	2.2 (2.7)	1.9 (2.3)	2.5 (2.8)	2.9 (3.0)	2.9 (3.1)	2.0 (2.9)
Debut age of diabetes (years) - mean (SD)	44.5 (6.8)	44.8 (6.9)	44.6 (6.6)	44.0 (6.7)	43.8 (6.8)	44.1 (7.3)
HbA$_{1c}$ (mmol/mol)[a]- mean (SD)	56.4 (17.7)	44.1 (5.3)	56.6 (2.6)	67.0 (2.9)	77.1 (2.9)	98.3 (12.9)
HbA$_{1c}$ (%)[a]- mean (SD)	7.3 (0.67)	6.2 (0.48)	7.3 (0.24)	8.3 (0.27)	9.2 (0.27)	11.1 (1.18)
LDL cholesterol (mmol/L) - mean (SD)	3.1 (1.0)	3.0 (0.9)	3.1 (1.0)	3.1 (1.0)	3.1 (1.0)	3.3 (1.1)
Total cholesterol (mmol/L) - mean (SD)	5.2 (1.1)	5.1 (1.1)	5.2 (1.1)	5.3 (1.2)	5.4 (1.2)	5.7 (1.4)
Smokers - n (%)	14378 (23.9)	6950 (21.7)	2874 (25.0)	1877 (26.5)	1190 (28.6)	1487 (28.2)
Body Mass Index (kg/m^2) - mean (SD)	31.6 (6.3)	31.4 (6.1)	32.0 (6.4)	31.9 (6.2)	32.2 (6.5)	31.5 (6.5)
Systolic BP (mmHg) - mean (SD)	132.1 (16.1)	130.9 (15.7)	132.9 (16.2)	133.4 (16.3)	134.5 (17.2)	133.7 (17.0)
Diastolic BP (mmHg) - mean (SD)	81.0 (10.0)	80.3 (9.8)	81.4 (10.0)	81.7 (10.1)	82.3 (10.5)	82.8 (10.5)
Albuminuria - n (%)						
No albuminuria	40386 (83.7)	22229 (86.2)	7863 (83.7)	4598 (80.0)	2598 (78.5)	3098 (76.8)
Microalbuminuria	5594 (11.6)	2550 (9.9)	1087 (11.6)	798 (13.9)	514 (15.5)	645 (16.0)
Macroalbuminuria	2287 (4.7)	1005 (3.9)	442 (4.7)	348 (6.1)	199 (6.0)	293 (7.3)
eGFR (mL/min) - mean (SD)	97.2 (23.4)	94.0 (21.7)	97.5 (23.0)	100.3 (24.1)	102.8 (25.6)	108.0 (27.1)
Antihypertensives - n (%)	24132 (39.6)	13163 (40.5)	4870 (41.5)	2825 (39.3)	1591 (37.8)	1683 (31.6)
Statins - n (%)	17970 (29.4)	9404 (28.8)	3745 (31.7)	2236 (31.1)	1230 (29.1)	1355 (25.4)
Diabetes treatment - n (%)						
No pharmacologic treatment	22913 (34.8)	16627 (47.6)	3176 (25.1)	1352 (17.4)	696 (15.2)	1062 (18.0)
Oral agents	31742 (48.2)	14264 (40.8)	7200 (57.0)	4446 (57.3)	2612 (57.0)	3220 (54.4)
Insulin	6345 (9.6)	2561 (7.3)	1272 (10.1)	1045 (13.5)	678 (14.8)	789 (13.3)
Insulin + oral agents	4865 (7.4)	1514 (4.3)	990 (7.8)	918 (11.8)	599 (13.1)	844 (14.3)

Patients 55-74 years of age

	**≤52 mmol/mol (≤6.9%)**	**53-62 mmol/mol (7.0-7.8%)**	**63-72 mmol/mol (7.9-8.7%)**	**73-82 mmol/mol (8.8-9.7%)**	**≥83 mmol/mol (≥9.7%)**	**≤52 mmol/mol (≤6.9%)**
Age (years) - mean (SD)	64.1 (5.4)	64.4 (5.3)	64.1 (5.4)	63.5 (5.4)	63.1 (5.4)	62.7 (5.2)
Diabetes duration (years) - mean (SD)	2.9 (3.0)	2.4 (2.7)	3.5 (3.2)	4.0 (3.4)	3.9 (3.5)	2.6 (3.3)

Debut age of diabetes (years) - mean (SD)	61.2 (6.0)	61.9 (5.8)	60.5 (6.1)	59.6 (6.2)	59.3 (6.3)	60.2 (6.2)
HbA1c (mmol/mol)[a] - mean (SD)	53.2 (14.6)	44.7 (4.9)	56.4 (2.6)	66.8 (2.8)	76.8 (2.8)	98.1 (13.1)
HbA1c (%)[a] - mean (SD)	7.0 (1.34)	6.2 (0.45)	7.3 (0.24)	8.3 (0.26)	9.2 (0.26)	11.1 (1.20)
LDL cholesterol (mmol/L) - mean (SD)	3.0 (1.0)	3.0 (0.9)	3.0 (1.0)	3.0 (1.0)	3.0 (1.0)	3.3 (1.1)
Total cholesterol (mmol/L) - mean (SD)	5.1 (1.1)	5.1 (1.1)	5.1 (1.1)	5.2 (1.1)	5.3 (1.2)	5.6 (1.3)
Smokers - n (%)	25194 (16.8)	14578 (15.9)	5189 (17.0)	2631 (18.5)	1309 (19.6)	1487 (20.6)
Body Mass Index (kg/m^2) - mean (SD)	30.0 (5.1)	29.8 (5.1)	30.3 (5.2)	30.4 (5.2)	30.5 (5.4)	30.2 (5.5)
Systolic BP (mmHg) - mean (SD)	140.1 (17.1)	139.1 (16.7)	141.0 (17.2)	141.9 (17.6)	142.6 (18.3)	142.0 (18.8)
Diastolic BP (mmHg) - mean (SD)	79.7 (9.4)	79.2 (9.3)	80.0 (9.5)	80.6 (9.5)	81.3 (9.6)	82.3 (10.2)
Albuminuria - n (%)						
No albuminuria	99060 (82.0)	62154 (84.6)	19829 (80.1)	8965 (77.3)	3982 (74.3)	4130 (73.7)
Microalbuminuria	15277 (12.6)	8112 (11.0)	3433 (13.9)	1810 (15.6)	927 (17.3)	995 (17.8)
Macroalbuminuria	6471 (5.4)	3214 (4.4)	1503 (6.1)	825 (7.1)	451 (8.4)	478 (8.5)
eGFR (mL/min) - mean (SD)	82.9 (20.7)	81.5 (19.6)	83.3 (20.9)	84.9 (22.1)	87.1 (23.3)	92.0 (24.9)
Antihypertensives - n (%)	102195 (66.8)	64163 (68.7)	20586 (66.2)	9319 (63.8)	4084 (60.4)	4043 (56.0)
Statins - n (%)	69106 (45.1)	43218 (46.2)	14418 (46.3)	6296 (43.3)	2706 (40.3)	2468 (34.3)
Diabetes treatment - n (%)						
No pharmacologic treatment	64451 (39.5)	51994 (52.4)	8246 (24.9)	2274 (14.6)	874 (12.1)	1063 (13.3)
Oral agents	73598 (45.1)	38311 (38.6)	18538 (56.0)	8645 (55.6)	3827 (52.9)	4277 (53.7)
Insulin	12220 (7.5)	4838 (4.9)	2883 (8.7)	2111 (13.6)	1167 (16.1)	1221 (15.3)
Insulin + oral agents	12878 (7.9)	4161 (4.2)	3434 (10.4)	2507 (16.1)	1373 (19.0)	1403 (17.6)
Patients ≥75 years of age						
	**Patients overall**	**≤52 mmol/mol (≤6.9%)**	**53-62 mmol/mol (7.0-7.8%)**	**63-72 mmol/mol (7.9-8.7%)**	**73-82 mmol/mol (8.8-9.7%)**	**≥83 mmol/mol (≥9.7%)**
Age (years) - mean (SD)	79.5 (3.9)	79.6 (3.9)	79.5 (3.9)	79.6 (3.9)	79.7 (4.0)	79.5 (3.9)
Diabetes duration (years) - mean (SD)	3.4 (3.2)	3.0 (3.0)	4.1 (3.3)	4.5 (3.4)	4.4 (3.4)	3.2 (3.5)
Debut age of diabetes (years) - mean (SD)	76.1 (4.9)	76.5 (4.7)	75.5 (5.1)	75.2 (5.3)	75.1 (5.1)	76.4 (5.5)
HbA1c (mmol/mol)[a] - mean (SD)	51.7 (12.2)	44.9 (4.8)	56.4 (2.5)	66.7 (2.8)	76.7 (2.9)	97.0 (13.0)
HbA1c (%)[a] - mean (SD)	6.9 (1.12)	6.3 (0.44)	7.3 (0.23)	8.3 (0.26)	9.2 (0.27)	11.0 (1.19)
LDL cholesterol (mmol/L) - mean (SD)	2.9 (1.0)	2.9 (0.9)	3.0 (1.0)	3.0 (1.0)	2.9 (1.0)	3.3 (1.0)
Total cholesterol (mmol/L) - mean (SD)	5.1 (1.1)	5.0 (1.1)	5.1 (1.1)	5.2 (1.1)	5.1 (1.1)	5.4 (1.2)

Smokers - n (%)	1965 (5.8)	1290 (5.9)	384 (5.3)	161 (5.4)	72 (6.3)	58 (6.6)
Body Mass Index (kg/m^2) - mean (SD)	28.1 (4.6)	27.9 (4.5)	28.5 (4.5)	28.5 (4.6)	28.7 (4.8)	28.4 (5.0)
Systolic BP (mmHg) - mean (SD)	145.5 (18.6)	144.7 (18.3)	146.5 (18.9)	147.6 (19.0)	147.9 (19.6)	147.1 (19.5)
Diastolic BP (mmHg) - mean (SD)	76.5 (9.6)	76.1 (9.5)	77.0 (9.7)	77.9 (9.7)	78.4 (9.9)	78.5 (9.7)
Albuminuria - n (%)						
No albuminuria	19643 (77.4)	12873 (79.0)	4124 (76.1)	1607 (72.6)	592 (71.9)	447 (71.9)
Microalbuminuria	3565 (14.0)	2177 (13.4)	779 (14.4)	375 (16.9)	139 (16.9)	95 (15.3)
Macroalbuminuria	2171 (8.6)	1253 (7.7)	515 (9.5)	231 (10.4)	92 (11.2)	80 (12.9)
eGFR (mL/min) - mean (SD)	67.8 (19.2)	67.8 (19.0)	67.4 (19.2)	68.0 (20.6)	68.2 (20.8)	70.4 (20.7)
Antihypertensives - n (%)	26924 (76.6)	17431 (77.7)	5811 (76.8)	2246 (73.3)	832 (69.9)	604 (67.1)
Statins - n (%)	12614 (36.2)	8391 (37.7)	2713 (36.2)	944 (31.4)	340 (29.0)	226 (25.7)
Diabetes treatment - n (%)						
No pharmacologic treatment	17271 (46.3)	13885 (58.3)	2498 (31.3)	608 (18.7)	156 (12.5)	124 (12.8)
Oral agents	15216 (40.8)	8075 (33.9)	4211 (52.7)	1793 (55.0)	671 (53.6)	466 (48.1)
Insulin	2687 (7.2)	1130 (4.7)	690 (8.6)	442 (13.6)	227 (18.1)	198 (20.4)
Insulin + oral agents	2119 (5.7)	731 (3.1)	593 (7.4)	417 (12.8)	197 (15.7)	181 (18.7)

[a]Categories of HbA1c was defined based on mmol/mol (IFCC) data. For exact category levels in % (DCCT) use the conversion formula, DCCT=0.09148*IFCC+2.152

***ESM Table 3*: Hospitalizations for heart failure in persons with diabetes type 2 and controls per 1,000 patient years at baseline by age and sex with 95% confidence intervals estimated by exact Poisson confidence limits**

**Group**	**Heart failure (any position)**	**Event rate/1,000 person years (95 % CI)**	**Heart failure (first two positions)**	**Event rate/1,000 person years (95 % CI)**
**Men overall**				
Controls	27344	6.2 (6.1, 6.3)	12305	2.8 (2.7,2.8)
Type 2 diabetes	10089	11.8 (11.5, 12.0)	5186	6.0 (5.8, 6.1)
**Women overall**				
Controls	22813	6.2 (6.2, 6.3)	9954	2.7 (2.6, 2.8)
Type 2 diabetes	8626	12.1 (11.9, 12.4)	4140	5.7 (5.6, 5.9)
**Men aged <55**				
Controls	1161	0.9 (0.9, 1.0)	483	0.4 (0.3, 0.4)
Type 2 diabetes	798	3.2 (3.0, 3.4)	404	1.6 (1.5, 1.8)
**Women aged <55**				
Controls	281	0.3 (0.3, 0.4)	117	0.1 (0.1, 0.2)
Type 2 diabetes	334	2.1 (1.9, 2.3)	160	1.0 (0.9, 1.2)
**Men aged 55-74**				
Controls	15583	5.6 (5.5, 5.7)	6679	2.4 (2.3, 2.4)
Type 2 diabetes	6208	11.5 (11.2, 11.8)	3176	5.8 (5.6, 6.0)
**Women aged 55-74**				
Controls	8651	3.9 (3.8, 4.0)	3533	1.6 (1.5, 1.6)
Type 2 diabetes	4120	9.6 (9.3, 9.9)	1923	4.4 (4.2, 4.6)
**Men aged ≥75**				
Controls	10600	29.8 (29.3, 30.4)	5143	14.0 (13.7, 14.4)
Type 2 diabetes	3083	43.7 (42.2, 45.3)	1606	21.7 (20.7, 22.8)
**Women aged ≥75**				
Controls	13881	22.3 (22.0, 22.7)	6304	9.9 (9.6, 10.1)
Type 2 diabetes	4172	34.3 (33.2, 35.3)	2057	16.2 (15.5, 16.9)

Events are presented as numbers. Incidence rates are presented as mean and standard deviation.

ESM Table 4: **Hospitalizations for heart failure in persons with diabetes type 2 per 1,000 patient years at baseline by age and HbA_{1c} mmol/mol (%) with 95% confidence intervals estimated by exact Poisson confidence limits**

Group	Events (heart failure in any position)	Event rate/1,000 person years (95 % CI)	Events (heart failure the first two positions)	Event rate/1,000 person years (95 % CI)
**Overall**				
Controls	50157	6.2 (6.2, 6.3)	22259	2.7 (2.7,2.8)
≤52 (≤6.9)	9860	10.8 (10.6, 11.0)	4784	5.2 (5.0, 5.3)
53-62 (7.0-7.8)	4395	13.4 (13.0, 13.8)	2204	6.6 (6.3, 6.9)
63-72 (7.9-8.7)	2336	13.8 (13.2, 14.3)	1208	7.0 (6.6, 7.4)
73-82 (8.8-9.7)	1169	14.5 (13.7, 15.4)	599	7.3 (6.7, 7.9)
≥83 (≥9.7)	955	12.5 (11.8, 13.4)	531	6.9 (6.3, 7.5)
**Age <55**				
Controls	1442	0.7 (0.7, 0.7)	600	0.3 (0.3, 0.3)
≤52 (≤6.9)	422	2.0 (1.8, 2.2)	207	1.0 (0.8, 1.1)
53-62 (7.0-7.8)	244	3.0 (2.7, 3.4)	130	1.6 (1.3, 1.9)
63-72 (7.9-8.7)	184	3.5 (3.0, 4.1)	78	1.5 (1.2, 1.9)
73-82 (8.8-9.7)	135	4.6 (3.9, 5.5)	63	2.1 (1.6, 2.7)
≥83 (≥9.7)	147	4.8 (4.0, 5.6)	86	2.8 (2.2, 3.4)
**Age 55-74**				
Controls	24234	4.8 (4.8, 4.9)	10212	2.0 (2.0, 2.1)
≤52 (≤6.9)	5222	9.1 (8.8, 9.3)	2442	4.2 (4.0, 4.4)
53-62 (7.0-7.8)	2380	11.6 (11.1, 12.0)	1186	5.7 (5.4, 6.0)
63-72 (7.9-8.7)	1373	13.6 (12.9, 14.4)	732	7.1 (6.6, 7.7)
73-82 (8.8-9.7)	736	16.3 (15.2, 17.6)	395	8.6 (7.8, 9.5)
≥83 (≥9.7)	617	15.0 (13.9, 16.3)	344	8.2 (7.4, 9.1)
**Age ≥75**				
Controls	24481	25.1 (24.7, 25.4)	11447	11.4 (11.2, 11.6)
≤52 (≤6.9)	4216	34.3 (33.3, 35.4)	2135	16.7 (16.0, 17.4)
53-62 (7.0-7.8)	1771	42.5 (40.5, 44.5)	888	20.3 (19.0, 21.7)
63-72 (7.9-8.7)	779	45.7 (42.5, 49.0)	398	22.2 (20.1, 24.5)
73-82 (8.8-9.7)	298	47.3 (42.1, 53.0)	141	21.0 (17.7, 24.8)
≥83 (≥9.7)	191	43.5 (37.5, 50.1)	101	21.9 (17.8, 26.6)

Events are presented as numbers. Incidence rates are presented as mean and standard deviation.

***ESM Table 5*: Hospitalizations for heart failure in persons with diabetes type 2 per 1,000 patient years at baseline by age and presence of albuminuria with 95% confidence intervals estimated by exact Poisson confidence limits**

Group	Events (heart failure in any position)	Event rate/1,000 person years (95 % CI)	Events (heart failure the first two positions)	Event rate/1,000 person years (95 % CI)
**Overall**				
Controls	50157	6.2 (6.2, 6.3)	22259	2.7 (2.7, 2.8)
No albuminuria	9691	9.9 (9.7, 10.1)	4769	4.8 (4.7, 5.0)
Microalbuminuria	2386	16.4 (15.7, 17.0)	1269	8.5 (8.1, 9.0)
Macroalbuminuria	1566	25.4 (24.1, 26.7)	821	12.9 (12.1, 13.9)
Stage 5 CKD	70	31.9 (24.9, 40.3)	28	12.3 (8.2, 17.8)
**Age <55**				
Controls	1442	0.7 (0.7, 0.7)	600	0.3 (0.3, 0.3)
No albuminuria	625	2.4 (2.2, 2.6)	299	1.1 (1.0, 1.3)
Microalbuminuria	158	4.5 (3.8, 5.2)	80	2.2 (1.8, 2.8)
Macroalbuminuria	94	6.7 (5.4, 8.2)	50	3.5 (2.6, 4.6)
Stage 5 CKD	6	11.1 (4.1, 24.2)	2	3.7 (0.4, 13.3)
**Age 55-74**				
Controls	24234	4.8 (4.8, 4.9)	10212	2.0 (2.0, 2.1)
No albuminuria	5468	9.0 (8.7, 9.2)	2663	4.3 (4.1, 4.5)
Microalbuminuria	1411	15.3 (14.5, 16.1)	738	7.8 (7.3, 8.4)
Macroalbuminuria	884	23.6 (22.0, 25.2)	442	11.5 (10.4, 12.6)
Stage 5 CKD	49	32.7 (24.2, 43.3)	19	12.1 (7.3, 18.9)
**Age ≥75**				
Controls	24481	25.1 (24.7, 25.4)	11447	11.4 (11.2, 11.6)
No albuminuria	3598	33.5 (32.4, 34.6)	1807	16.2 (15.4, 16.9)
Microalbuminuria	817	45.2 (42.1, 48.4)	451	23.8 (21.7, 26.1)
Macroalbuminuria	588	58.1 (53.5, 63.0)	329	30.8 (27.6, 34.3)
Stage 5 CKD	15	95.3 (53.3, 157.2)	7	42.4 (17.1, 87.5)

Events are presented as numbers. Incidence rates are presented as mean and standard deviation.

***ESM Table 6*: Hospitalizations for heart failure in persons with diabetes type 2 per 1,000 patient years at baseline by age and eGFR (mL/min) with 95% confidence intervals estimated by exact Poisson confidence limits**

**Group**	**Events (heart failure in any position)**	**Event rate/1,000 person years (95 % CI)**	**Events (heart failure the first two positions)**	**Event rate/1,000 person years (95 % CI)**
**Overall**				
Controls	50157	6.2 (6.2, 6.3)	22259	2.7 (2.7, 2.8)
>90	2511	6.3 (6.1, 6.6)	1173	2.9 (2.8, 3.1)
>60-90	6428	10.1 (9.9,10.3)	3169	4.9 (4.7, 5.1)
>45-60	2604	21.1 (20.3, 21.9)	1294	10.2 (9.7, 10.8)
>30-45	989	41.4 (38.9, 44.1)	513	20.6 (18.8, 22.4)
15-30	219	65.9 (57.5, 75.2)	123	35.2 (29.2, 42.0)
Stage 5 CKD	70	31.9 (24.9, 40.3)	28	12.3 (8.2, 17.8)
**Age <55**				
Controls	1442	0.7 (0.7, 0.7)	600	0.3 (0.3, 0.3)
>90	333	2.1 (1.9, 2.4)	162	1.0 (0.9, 1.2)
>60-90	295	2.4 (2.1, 2.6)	135	1.1 (0.9, 1.3)
>45-60	31	3.8 (2.6, 5.4)	14	1.7 (0.9, 2.9)
>30-45	11	13.4 (6.7, 23.9)	6	7.1 (2.6, 15.5)
15-30	6	29.5 (10.8, 64.3)	2	9.3 (1.1, 33.6)
Stage 5 CKD	6	11.1 (4.1, 24.2)	2	3.7 (0.4,13.3)
**Age 55,74**				
Controls	24234	4.8 (4.8, 4.9)	10212	2.0 (2.0, 2.1)
>90	1712	7.7 (7.4, 8.1)	770	3.4 (3.2, 3.7)
>60-90	3695	8.6 (8.3, 8.8)	1804	4.1 (3.9, 4.3)
>45-60	1045	13.8 (12.9,14.6)	531	6.9 (6.3, 7.5)
>30-45	324	27.8 (24.8, 31.0)	163	13.5 (11.5, 15.8)
15-30	78	45.8 (36.2, 57.2)	38	21.5 (15.2, 29.5)
Stage 5 CKD	49	32.7 (24.2, 43.3)	19	12.1 (7.3, 18.9)
**Age ≥75**				
Controls	24481	25.1 (24.7, 25.4)	11447	11.4 (11.2, 11.6)
>90	466	28.2 (25.7, 30.9)	241	14.2 (12.5, 16.1)
>60-90	2438	30.4 (29.2, 31.6)	1230	14.9 (14.0, 15.7)
>45-60	1528	39.0 (37.0, 41.0)	749	18.3 (17.0, 19.6)
>30-45	654	57.3 (53.0, 61.9)	344	28.5 (25.5, 31.7)
15-30	135	95.3 (79.9, 112.8)	83	54.9 (43.7, 68.0)
Stage 5 CKD	15	95.3 (53.3, 157.2)	7	42.4 (17.1, 87.5)

Events are presented as numbers. Incidence rates are presented as mean and standard deviation.

***ESM Table 7*: Baseline (Patients included vs. Patients with missing HbA_{1c} after exclusion criteria)**

	**Patients included, overall**	**Patients with missing HbA1c**
Individuals - n	266305	9963
Women - n (%)	120641 (45.3)	4366 (43.8)
Age (years) - mean (SD)	62.0 (11.6)	59.7 (14.5)
Atrial fibrillation - n (%)	11078 (4.2)	515 (5.2)
Myocardial infarction - n (%)	16765 (6.3)	700 (7.0)
Coronary heart disease - n (%)	32637 (12.3)	1344 (13.5)
Stroke - n (%)	12262 (4.6)	500 (5.0)
Cancer - n (%)	16814 (6.3)	667 (6.7)
Dementia - n (%)	495 (0.2)	45 (0.5)
Renal dialysis or transplantation - n (%)	394 (0.1)	32 (0.3)
Diabetes duration (years) - mean (SD)	2.8 (3.0)	3.0 (3.1)
Debut age of diabetes (years) - mean (SD)	59.2 (11.5)	56.7 (14.8)
HbA_{1c} (mmol/mol)[a] - mean (SD)	53.8 (15.2)	NA
HbA_{1c} (%)[a] - mean (SD)	7.1 (1.4)	NA
LDL cholesterol (mmol/L) - mean (SD)	3.0 (1.0)	3.0 (1.0)
Total cholesterol (mmol/L) - mean (SD)	5.1 (1.1)	5.2 (1.2)
Smokers - n (%)	41537 (17.0)	1172 (22.2)
Body Mass Index (kg/m^2) - mean (SD)	30.2 (5.5)	30.1 (5.9)
Systolic BP (mmHg) - mean (SD)	138.9 (17.6)	139.3 (19.1)
Diastolic BP (mmHg) - mean (SD)	79.6 (9.7)	80.0 (10.2)
Albuminuria - n (%)		
No albuminuria	159089 (81.8)	2829 (77.6)
Microalbuminuria	24436 (12.6)	574 (15.7)
Macroalbuminuria	10929 (5.6)	242 (6.6)
eGFR (mL/min)	84.3 (23.0)	84.6 (26.7)
Antihypertensives - n (%)	153251 (61.5)	4903 (54.4)
Statins - n (%)	99690 (40.0)	3410 (37.7)
Diabetes treatment - n (%)		
No pharmacologic treatment	104635 (39.3)	4570 (45.9)
Oral agents	120556 (45.3)	3590 (36.0)
Insulin	21252 (8.0)	944 (9.5)
Insulin + oral agents	19862 (7.5)	859 (8.6)

[a]Categories of HbA1c was defined based on mmol/mol (IFCC) data. For exact category levels in % (DCCT) use the conversion formula, DCCT=0.09148*IFCC+2.152

Original cohort

Patients with type 2 diabetes, n=457,473
Controls, n= 2,287,365

Exclude controls with inconsistent vital data (negative survival).

Controls excluded, n=26,215

Cohort left after exclusion of inconsistent vital data

Patients with type 2 diabetes, n=457,473
Controls, n= 2,261,150

Exclude the complete matched set (1 patient & 5 controls) if a patient or control had previous heart failure (in any diagnostic position).

Patients excluded, n=85,078
Controls excluded, n=411,415

Cohort left without heart failure at baseline

Patients with type 2 diabetes, 372,395
Controls, n= 1,849,735

Exclude the complete matched set (1 patient & 5 controls if a patient had missing value of diabetes duration at baseline.

Patients excluded, n=40,285
Controls excluded, n=199,767

Cohort left without missing diabetes duration at baseline

Patients with type 2 diabetes, n=332,110
Controls, n= 1,649,968

Exclude the complete matched set (1 patient & 5 controls) if a patient had diabetes duration >10 years at baseline.

Patients excluded, n=55,842
Controls excluded, n=276,964

Cohort left with diabetes duration ≤10 years

Patients with type 2 diabetes, n=276 ,268
Controls, n= 1,373,004

Exclude the complete matched set (1 patient & 5 controls) if missing value of HbA_{1c} at baseline.

Patients excluded, n=9,963
Controls excluded, n=49,500

Cohort left with imputed HbA_{1c} at baseline

Patients with type 2 diabetes, n=266,305
Controls, n= 1,323,504

ESM Fig. 1

Flow-chart for the final sample. Cohort with exclusion of patients with diabetes duration >10 years, or missing diabetes duration at baseline

Groups by HbA_{1c} mmol/mol (%)	Events	Model 1 HR (CI 95%)	Model 2 HR (CI 95%)
≤52 (≤6.9)	9860	1.38 (1.33, 1.43)	1.23 (1.18, 1.28)
53-62 (7.0-7.8)	4395	1.69 (1.61, 1.77)	1.49 (1.42, 1.56)
63-72 (7.9-8.7)	2336	1.96 (1.86, 2.07)	1.69 (1.60, 1.79)
73-82 (8.8-9.7)	1169	2.46 (2.30, 2.63)	2.13 (1.99, 2.28)
≥83 (≥9.7)	955	2.72 (2.53, 2.91)	2.37 (2.20, 2.54)

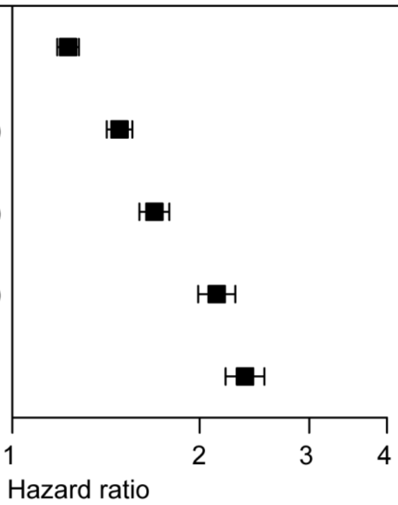

ESM Fig. 2

HR (CI 95%) for the risk of hospitalization for heart failure among patients with type 2 diabetes by HbA_{1c} mmol/mol (%), compared to age- and sex matched controls from the general population. Model 1 shows HRs adjusted for age, sex and duration of diabetes. Model 2 shows HRs adjusted for age, sex, duration of diabetes, income, education, marital status, immigrant status, stroke, acute myocardial infarction, coronary heart disease, atrial fibrillation and renal dialysis or transplantation. HbA_{1c} is given as mmol/mol (International Federation of Clinical Chemistry) and % (Diabetes Control and Complications Trial). Black boxes indicate HRs while error bars are CIs 95%. Plots for Model 1 are not shown. Note that HR are plotted on a log scale.

Groups by albuminuria and Stage 5 CKD	Events	Model 1 HR (CI 95%)	Model 2 HR (CI 95%)
No albuminuria	9691	1.38 (1.31, 1.44)	1.23 (1.18, 1.29)
Microalbuminuria	2386	2.03 (1.92, 2.16)	1.75 (1.65, 1.86)
Macroalbuminuria	1566	2.62 (2.46, 2.80)	2.18 (2.04, 2.34)
Stage 5 CKD	70	5.44 (4.29, 6.91)	4.18 (3.28, 5.32)

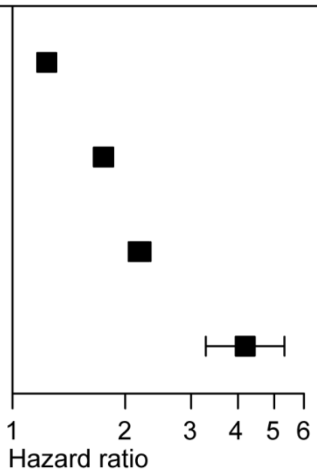

ESM Fig. 3

HR (CI 95%) for the risk of hospitalization for heart failure among patients with type 2 diabetes by albuminuria and Stage 5 CKD, compared to age- and sex matched controls from the general population. Model 1 shows HRs adjusted for age, sex and duration of diabetes. Model 2 shows HRs adjusted for age, sex, duration of diabetes, income, education, marital status, immigrant status, stroke, acute myocardial infarction, coronary heart disease and atrial fibrillation. Black boxes indicate HRs while error bars are CIs 95%. Plots for Model 1 are not shown. Note that HR are plotted on a log scale.

Groups by eGFR (mL/min) and Stage 5 CKD	Events	Model 1 HR (CI 95%)	Model 2 HR (CI 95%)
>90	2511	1.63 (1.54, 1.71)	1.46 (1.38, 1.54)
>60-90	6428	1.38 (1.32, 1.45)	1.23 (1.18, 1.29)
>45-60	2604	1.60 (1.52, 1.69)	1.38 (1.30, 1.45)
>30-45	989	2.31 (2.15, 2.49)	1.86 (1.73, 2.00)
15-30	219	4.05 (3.52, 4.65)	2.98 (2.59, 3.43)
Stage 5 CKD	70	5.70 (4.49, 7.23)	4.40 (3.46, 5.60)

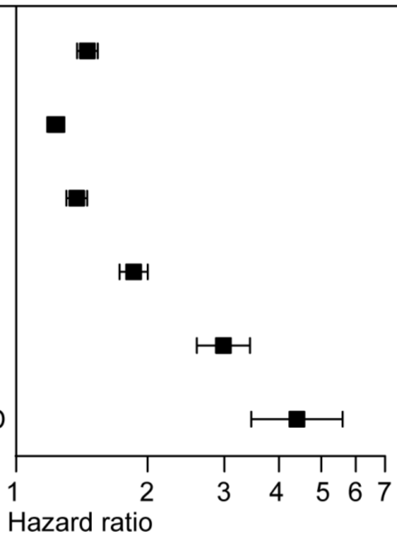

ESM Fig. 4

Hazard ratio (HR) for the risk of hospitalization for heart failure among patients with type 2 diabetes by eGFR (mL/min) and Stage 5 CKD, compared to age- and sex matched controls from the general population. Model 1 shows HRs adjusted for age, sex and duration of diabetes. Model 2 shows HRs adjusted for age, sex, duration of diabetes, income, education, marital status, immigrant status, stroke, acute myocardial infarction, coronary heart disease and atrial fibrillation. Black boxes indicate HRs while error bars are CIs 95%. Plots for Model 1 are not shown. Note that HR are plotted on a log scale.

eGFR (mL/min) and Stage 5 CKD by age group (years)	Events	Model 1 HR (CI 95%)	Model 2 HR (CI 95%)
Age <55			
>90	333	3.05 (2.55, 3.66)	2.36 (1.96, 2.83)
>60-90	295	2.93 (2.43, 3.53)	2.32 (1.92, 2.81)
>45-60	31	4.89 (3.33, 7.18)	3.77 (2.55, 5.58)
>30-45	11	15.21 (8.24, 28.10)	9.60 (4.88, 18.89)
15-30	6	36.00 (15.87, 81.69)	35.09 (15.45, 79.69)
Stage 5 CKD	6	12.95 (5.72, 29.29)	9.81 (4.33, 22.26)
Age 55-74			
>90	1712	1.78 (1.66, 1.91)	1.52 (1.42, 1.63)
>60-90	3695	1.59 (1.50, 1.69)	1.35 (1.27, 1.44)
>45-60	1045	2.14 (1.97, 2.31)	1.72 (1.59, 1.87)
>30-45	324	3.92 (3.48, 4.42)	2.95 (2.61, 3.33)
15-30	78	6.82 (5.43, 8.57)	5.23 (4.14, 6.60)
Stage 5 CKD	49	6.85 (5.15, 9.11)	4.56 (3.42, 6.08)
Age ≥75			
>90	466	1.03 (0.92, 1.15)	0.96 (0.85, 1.07)
>60-90	2438	1.06 (0.99, 1.14)	0.98 (0.91, 1.05)
>45-60	1528	1.28 (1.18, 1.38)	1.14 (1.05, 1.23)
>30-45	654	1.79 (1.62, 1.98)	1.51 (1.37, 1.67)
15-30	135	3.02 (2.52, 3.62)	2.34 (1.95, 2.81)
Stage 5 CKD	15	3.32 (1.99, 5.54)	2.78 (1.64, 4.71)

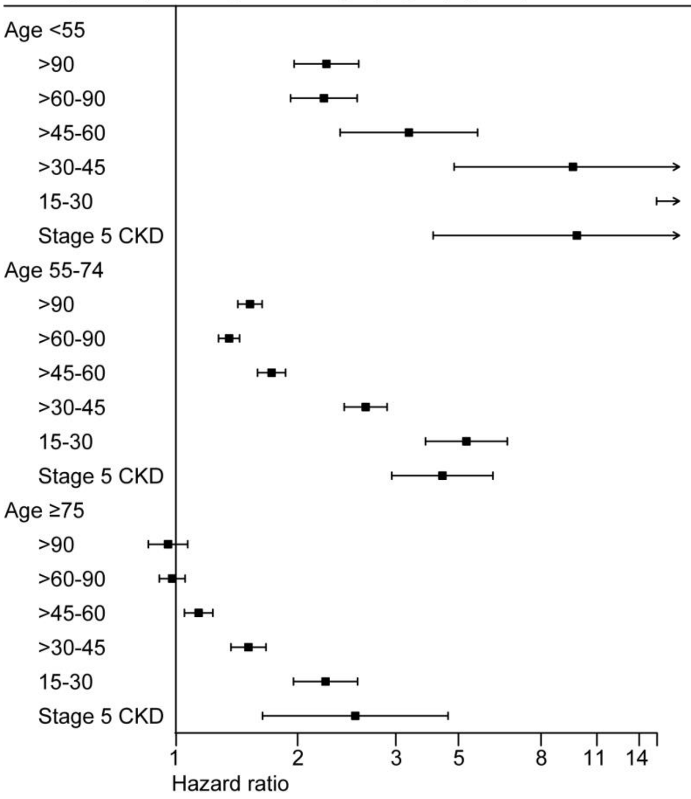

ESM Fig. 5

Hazard ratio (HR) for the risk of hospitalization for heart failure among patients with type 2 diabetes by eGFR (mL/min) and Stage 5 CKD by age group, compared to age- and sex matched controls from the general population. Model 1 shows HRs adjusted for age, sex and duration of diabetes. Model 2 shows HRs adjusted for age, sex, duration of diabetes, income, education, marital status, immigrant status, stroke, acute myocardial infarction, coronary heart disease and atrial fibrillation. Black boxes indicate HRs while error bars are CIs 95%. Plots for Model 1 are not shown. Note that HR are plotted on a log scale.

HbA1c mmol/mol (%) by age group (years)	Events	Model 1 HR (CI 95%)	Model 2 HR (CI 95%)
Age <55			
≤52 (≤6.9)	251	2.09 (1.68, 2.61)	1.70 (1.36, 2.12)
53-62 (7.0-7.8)	137	2.74 (2.12, 3.53)	2.09 (1.61, 2.71)
63-72 (7.9-8.7)	90	2.74 (2.06, 3.64)	2.02 (1.52, 2.70)
73-82 (8.8-9.7)	72	4.08 (3.02, 5.51)	3.13 (2.31, 4.25)
≥83 (≥9.7)	75	5.22 (3.94, 6.92)	4.05 (3.03, 5.40)
Age 55-74			
≤52 (≤6.9)	2844	1.36 (1.27, 1.47)	1.20 (1.11, 1.29)
53-62 (7.0-7.8)	1241	1.66 (1.52, 1.81)	1.44 (1.32, 1.58)
63-72 (7.9-8.7)	739	2.09 (1.89, 2.30)	1.81 (1.64, 2.00)
73-82 (8.8-9.7)	368	2.62 (2.31, 2.96)	2.25 (1.99, 2.55)
≥83 (≥9.7)	288	2.71 (2.38, 3.08)	2.24 (1.96, 2.55)
Age ≥75			
≤52 (≤6.9)	2151	0.95 (0.86, 1.05)	0.88 (0.80, 0.97)
53-62 (7.0-7.8)	869	1.13 (1.01, 1.26)	1.03 (0.92, 1.15)
63-72 (7.9-8.7)	365	1.19 (1.04, 1.36)	1.05 (0.91, 1.20)
73-82 (8.8-9.7)	135	1.30 (1.07, 1.57)	1.13 (0.93, 1.38)
≥83 (≥9.7)	80	1.19 (0.95, 1.50)	1.06 (0.83, 1.35)

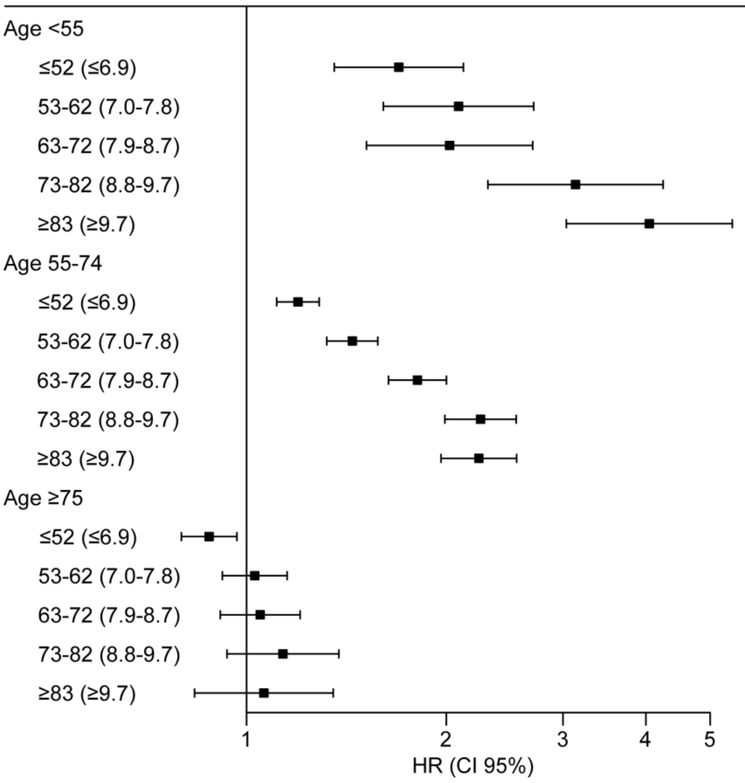

ESM Fig. 6

Hazard ratio (HR) for the risk of hospitalization for heart failure among patients with type 2 diabetes with normoalbuminuria by HbA_{1c} mmol/mol (%) by age group, compared to age- and sex matched controls from the general population. Model 1 shows HRs adjusted for age, sex and duration of diabetes. Model 2 shows HRs adjusted for age, sex, duration of diabetes, income, education, marital status, immigrant status, stroke, acute myocardial infarction, coronary heart disease, atrial fibrillation and renal dialysis or transplantation. HbA_{1c} is given as mmol/mol (International Federation of Clinical Chemistry) and % (Diabetes Control and Complications Trial). Black boxes indicate HRs while error bars are CIs 95%. Plots for Model 1 are not shown. Note that HR are plotted on a log scale.

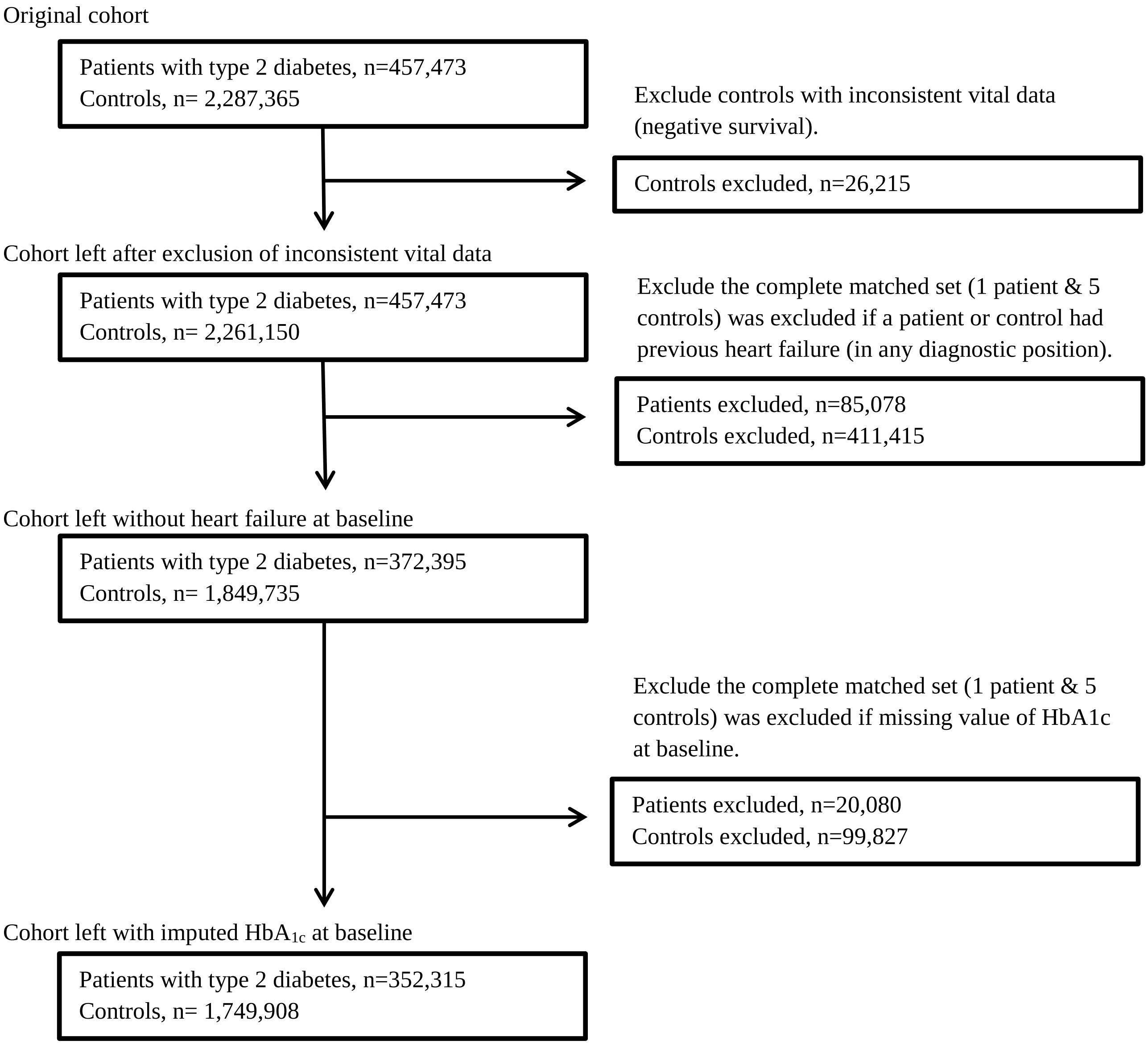

ESM Fig. 7

Second flow-chart. Cohort without exclusion of patients with missing diabetes duration at baseline and diabetes duration >10 years

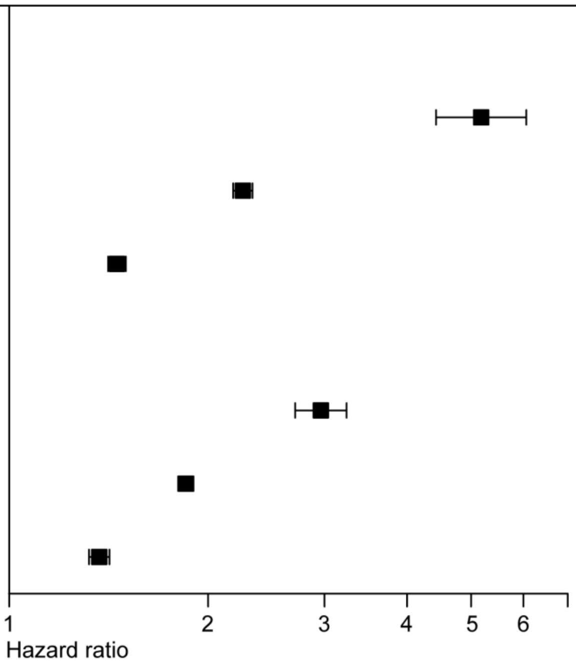

Age groups (years) by sex	Events	Model 1 HR (CI 95%)	Model 2 HR (CI 95%)
Women			
Age <55	442	6.78 (5.86, 7.84)	5.18 (4.43, 6.06)
Age 55-74	6495	2.68 (2.59, 2.77)	2.26 (2.18, 2.33)
Age ≥75	6939	1.61 (1.56, 1.66)	1.46 (1.41, 1.50)
Men			
Age <55	1019	3.88 (3.57, 4.22)	2.96 (2.71, 3.24)
Age 55-74	9668	2.21 (2.15, 2.26)	1.85 (1.80, 1.90)
Age ≥75	5051	1.49 (1.44, 1.55)	1.37 (1.32, 1.42)

ESM Fig. 8

Hazard ratio (HR) for the risk of hospitalization for heart failure among patients with type 2 diabetes by age group, compared to age- and sex matched controls from the general population. Model 1 shows HRs adjusted for age, sex and duration of diabetes. Model 2 shows HRs adjusted for age, sex, duration of diabetes, income, education, marital status, immigrant status, stroke, acute myocardial infarction, coronary heart disease, atrial fibrillation and renal dialysis or transplantation. Black boxes indicate HRs while error bars are CIs 95%. Plots for Model 1 are not shown. Analyses performed without exclusion of patients with missing variable (duration of diabetes) and longer diabetes duration >10 years. Note that HR are plotted on a log scale.

HbA$_{1c}$ (mmol/mol)/ (%) by age group (years)	Events	Model 1 HR (CI 95%)	Model 2 HR (CI 95%)
Age <55			
≤52 (≤6.9)	517	3.32 (2.99, 3.68)	2.62 (2.35, 2.91)
53-62 (7.0-7.8)	301	4.30 (3.78, 4.88)	3.26 (2.85, 3.72)
63-72 (7.9-8.7)	249	4.89 (4.25, 5.63)	3.70 (3.20, 4.27)
73-82 (8.8-9.7)	188	6.76 (5.77, 7.91)	5.14 (4.38, 6.03)
≥83 (≥9.7)	206	8.34 (7.18, 9.69)	6.45 (5.54, 7.51)
Age 55-74			
≤52 (≤6.9)	6971	1.97 (1.92, 2.03)	1.67 (1.63, 1.72)
53-62 (7.0-7.8)	3793	2.38 (2.30, 2.47)	1.99 (1.92, 2.06)
63-72 (7.9-8.7)	2647	2.92 (2.80, 3.05)	2.46 (2.36, 2.57)
73-82 (8.8-9.7)	1508	3.70 (3.51, 3.91)	3.04 (2.88, 3.22)
≥83 (≥9.7)	1244	4.67 (4.41, 4.96)	3.82 (3.60, 4.05)
Age ≥75			
≤52 (≤6.9)	6133	1.42 (1.38, 1.46)	1.30 (1.27, 1.34)
53-62 (7.0-7.8)	3094	1.66 (1.60, 1.73)	1.51 (1.45, 1.57)
63-72 (7.9-8.7)	1659	1.82 (1.72, 1.92)	1.63 (1.55, 1.72)
73-82 (8.8-9.7)	682	2.02 (1.87, 2.19)	1.80 (1.66, 1.96)
≥83 (≥9.7)	422	2.19 (1.98, 2.42)	1.99 (1.79, 2.20)

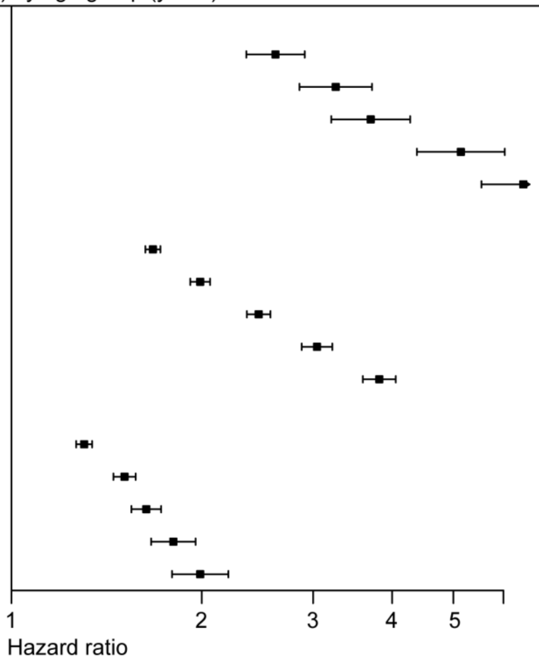

ESM Fig. 9

Hazard ratio (HR) for the risk of hospitalization for heart failure among patients with type 2 diabetes by HbA$_{1c}$ mmol/mol (%) by age group, compared to age- and sex matched controls from the general population. Model 1 shows HRs adjusted for age, sex and duration of diabetes. Model 2 shows HRs adjusted for age, sex, duration of diabetes, income, education, marital status, immigrant status, stroke, acute myocardial infarction, coronary heart disease, atrial fibrillation and renal dialysis or transplantation. HbA$_{1c}$ is given as mmol/mol (International Federation of Clinical Chemistry) and % (Diabetes Control and Complications Trial). Black boxes indicate HRs while error bars are CIs 95%. Plots for Model 1 are not shown. Analyses performed without exclusion of patients with missing variable (duration of diabetes) and longer diabetes duration >10 years. Note that HR are plotted on a log scale.

Albuminuria and Stage 5 CKD by age group (years)	Events	Model 1 HR (CI 95%)	Model 2 HR (CI 95%)
Age <55			
No albuminuria	780	3.74 (3.43, 4.09)	2.92 (2.66, 3.20)
Microalbuminuria	201	6.48 (5.56, 7.55)	4.76 (4.07, 5.58)
Macroalbuminuria	126	10.02 (8.31,12.08)	7.36 (6.08, 8.90)
Stage 5 CKD	11	16.15 (8.84, 29.50)	11.15 (6.06, 20.51)
Age 55-74			
No albuminuria	8167	1.99 (1.94, 2.04)	1.72 (1.68, 1.77)
Microalbuminuria	2381	3.19 (3.06, 3.34)	2.58 (2.46, 2.70)
Macroalbuminuria	1727	4.84 (4.60, 5.09)	3.69 (3.51, 3.89)
Stage 5 CKD	93	7.85 (6.35, 9.70)	5.36 (4.31, 6.68)
Age ≥75			
No albuminuria	5651	1.39 (1.35, 1.43)	1.29 (1.25, 1.33)
Microalbuminuria	1461	1.83 (1.73, 1.93)	1.64 (1.55, 1.74)
Macroalbuminuria	1134	2.39 (2.25, 2.55)	2.12 (1.99, 2.26)
Stage 5 CKD	40	3.86 (2.70, 5.50)	3.24 (2.25, 4.67)

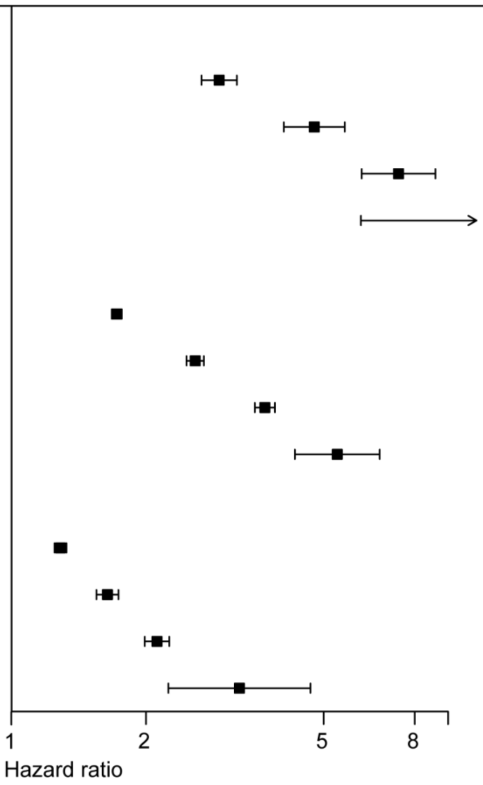

ESM Fig. 10

Hazard ratio (HR) for the risk of hospitalization for heart failure among patients with type 2 diabetes by albuminuria and Stage 5 CKD by age group, compared to age- and sex matched controls from the general population. Model 1 shows HRs adjusted for age, sex and duration of diabetes. Model 2 shows HRs adjusted for age, sex, duration of diabetes, income, education, marital status, immigrant status, stroke, acute myocardial infarction, coronary heart disease and atrial fibrillation. Black boxes indicate HRs while error bars are CIs 95%. Plots for Model 1 are not shown. Analyses performed without exclusion of patients with missing variable (duration of diabetes) and longer diabetes duration >10 years. Note that HR are plotted on a log scale.

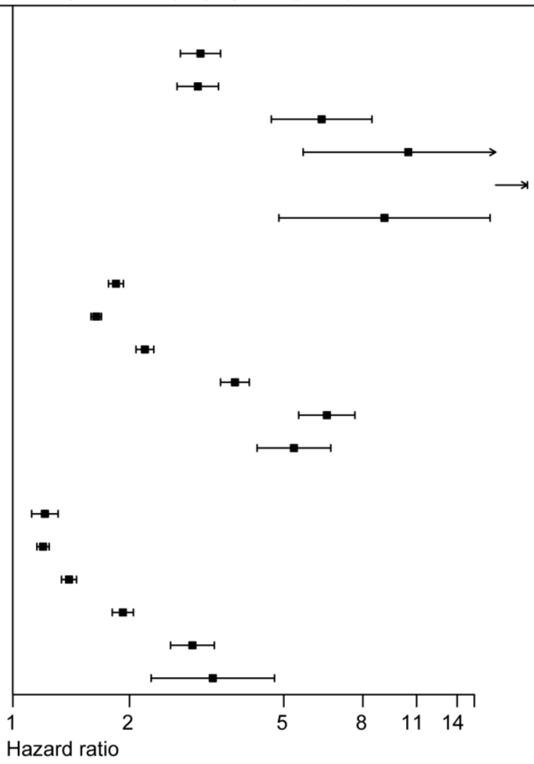

eGFR (mL/min) and Stage 5 CKD by age group (years)	Events	Model 1 HR (CI 95%)	Model 2 HR (CI 95%)
Age <55			
>90	429	4.03 (3.59, 4.52)	3.05 (2.71, 3.43)
>60-90	364	3.89 (3.45, 4.38)	3.00 (2.65, 3.39)
>45-60	47	8.21 (6.11, 11.02)	6.26 (4.64, 8.44)
>30-45	13	16.11 (9.11, 28.47)	10.47 (5.61, 19.55)
15-30	10	42.97 (22.12, 83.49)	41.26 (21.24, 80.16)
Stage 5 CKD	11	13.97 (7.58, 25.77)	9.09 (4.86, 17.02)
Age 55-74			
>90	2472	2.17 (2.08, 2.26)	1.85 (1.77, 1.93)
>60-90	5380	1.96 (1.90, 2.02)	1.64 (1.59, 1.69)
>45-60	1646	2.73 (2.59, 2.87)	2.19 (2.08, 2.31)
>30-45	596	5.02 (4.61, 5.46)	3.74 (3.44, 4.08)
15-30	158	8.16 (6.93, 9.61)	6.46 (5.46, 7.63)
Stage 5 CKD	93	7.77 (6.29, 9.61)	5.31 (4.27, 6.61)
Age ≥75			
>90	776	1.30 (1.20, 1.40)	1.21 (1.12, 1.31)
>60-90	3761	1.30 (1.25, 1.35)	1.20 (1.15, 1.24)
>45-60	2430	1.57 (1.50, 1.64)	1.40 (1.34, 1.46)
>30-45	1195	2.26 (2.13, 2.40)	1.92 (1.81, 2.05)
15-30	269	3.67 (3.22, 4.17)	2.91 (2.55, 3.31)
Stage 5 CKD	40	3.93 (2.76, 5.61)	3.28 (2.27, 4.73)

ESM Fig. 11

Hazard ratio (HR) for the risk of hospitalization for heart failure among patients with type 2 diabetes by eGFR (mL/min) and Stage 5 CKD by age group, compared to age- and sex matched controls from the general population. Model 1 shows HRs adjusted for age, sex and duration of diabetes. Model 2 shows HRs adjusted for age, sex, duration of diabetes, income, education, marital status, immigrant status, stroke, acute myocardial infarction, coronary heart disease and atrial fibrillation. Black boxes indicate HRs while error bars are CIs 95%. Plots for Model 1 are not shown. Analyses performed without exclusion of patients with missing variable (duration of diabetes) and longer diabetes duration >10 years. Note that HR are plotted on a log scale.
